# Differential Proteome Analysis of Bone Marrow Mesenchymal Stem Cells from Adolescent Idiopathic Scoliosis Patients

**DOI:** 10.1371/journal.pone.0018834

**Published:** 2011-04-22

**Authors:** Qianyu Zhuang, Jing Li, Zhihong Wu, Jianguo Zhang, Wei Sun, Tao Li, Yujuan Yan, Ying Jiang, Robert Chunhua Zhao, Guixing Qiu

**Affiliations:** 1 Department of Orthopedics, Peking Union Medical College Hospital, Beijing, People's Republic of China; 2 Center of Excellence in Tissue Engineering, Institute of Basic Medical Sciences and School of Basic Medicine, Chinese Academy of Medical Sciences and Peking Union Medical College, Beijing, People's Republic of China; 3 State Key Laboratory of Proteomics, Beijing Proteome Research Center, Beijing Institute of Radiation Medicine, Beijing, People's Republic of China; Instituto Nacional de Câncer, Brazil

## Abstract

Adolescent idiopathic scoliosis (AIS) is a complex three-dimensional deformity of the spine. The cause and pathogenesis of scoliosis and the accompanying generalized osteopenia remain unclear despite decades of extensive research. In this study, we utilized two-dimensional fluorescence difference gel electrophoresis (2D-DIGE) coupled with mass spectrometry (MS) to analyze the differential proteome of bone marrow mesenchymal stem cells (BM-MSCs) from AIS patients. In total, 41 significantly altered protein spots were detected, of which 34 spots were identified by MALDI-TOF/TOF analysis and found to represent 25 distinct gene products. Among these proteins, five related to bone growth and development, including pyruvate kinase M2, annexin A2, heat shock 27 kDa protein, γ-actin, and β-actin, were found to be dysregulated and therefore selected for further validation by Western blot analysis. At the protein level, our results supported the previous hypothesis that decreased osteogenic differentiation ability of MSCs is one of the mechanisms leading to osteopenia in AIS. In summary, we analyzed the differential BM-MSCs proteome of AIS patients for the first time, which may help to elucidate the underlying molecular mechanisms of bone loss in AIS and also increase understanding of the etiology and pathogenesis of AIS.

## Introduction

Adolescent idiopathic scoliosis (AIS) is a complex three-dimensional deformity of the spine occurring mostly in girls between 10 and 16 years of age during the pubertal growth spurt. The general incidence of AIS has been reported to range from 2.0% to 4.0% [1]–[3]. In Hong Kong, for instance, the prevalence rate of AIS increased from 2.7% in 1998 to 4.0% in 2003 among school children [4]. If untreated, severe scoliotic deformity affecting the thoracic region can progress, impairing cardiopulmonary function, thereby increasing the mortality rate [5], [6].

The main treatment of AIS includes full-time bracing [7], which may cause back pain along with psychological disorder, and pedical screw instrumentation, which inevitably leads to major operative trauma, decreased spinal range of motion [8], and even permanent catastrophic neurologic or vascular injury in case of screw malposition [9]. With the ultimate hope of developing more specific treatments and avoiding the above risks, extensive studies are carried out from different aspects, including histological, immunofluorescent, genetic and molecular biological studies of blood and tissue specimens from different animal models and AIS patients, as well as the imaging, cognitive, endocrinological, and neurological researches in AIS paitents [10]–[16]. However, no single factor can explain the whole clinical picture of AIS, and the pathogenesis remains largely unknown [17], [18].

The low bone mineral density (BMD) in AIS reported by many authors suggested the possibility of bone metabolism disturbance [19]–[25]. Moreover, a bone biopsy study reported diminished number of osteoblasts and a corresponding reduction in osteoclast number in patients with AIS [26], indicating disturbance in the bone metabolism as a primary change. In spite of the presence of much controversy engendered by all the hypotheses and considerable gaps in the related knowledge, there is a growing consensus that anomalies of bone growth and development are strongly related to the onset and progression of scoliosis.

Mesenchymal stem cells (MSCs) are well known for playing a pivotal role in bone growth, bone modeling, and bone remodeling. MSCs are able to act as a source of progenitors for osteoblasts, and also regulate osteoclastogenesis via their expression of RANKL and OPG [27]–[31]. Furthermore, MSCs are indispensable in both intramembranous and endochondral bone formation. Intramembranous bone formation requires periosteal MSCs to undergo osteogenic differentiation and form bone without a preceding cartilage step. Endochondral bone formation, running alongside the intramembranous process, begins with the differentiation of condensed MSCs into chondroprogenitors and osteoprogenitors, both of which will undergo coupled proliferation and differentiation programs and ultimately form mature cartilage and bone [27].

Given the functional characteristics of MSCs in bone formation and resorption, it is very likely that MSCs play a significant role in the etiology and pathogenesis of AIS. A recent study, which supports our speculation, observed lower osteogenic differentiation abilities and alkaline phosphatase activities of MSCs from AIS patients [32], indicating that the decreased osteogenic differentiation ability of MSCs might be a possible mechanism leading to low bone mass in AIS. However, to the best of the authors' knowledge, there have been virtually no reports on the proteomics of MSCs from AIS patients in the literature up to this point.

Therefore, to gain an insight into the pathogenesis of AIS, we studied specific alterations in the proteome of MSCs by using two-dimensional fluorescence difference gel electrophoresis (2D-DIGE)and mass spectrometry (MS). This study focused on depicting the differential proteome of bone marrow mesenchymal stem cells (BM-MSCs) from AIS patients, by comparing the MSCs proteomic profile of AIS patients with that of lower-leg fractured non-AIS patients.

## Methods

### Patients and Specimens

Bone marrow (BM) aspirates were obtained from six AIS patients (mean age 12.3 years, range 11–14) and six non-AIS patients with lower-leg fracture (mean age 12.6 years, range 11–14). In the AIS group, all of the patients underwent full clinical and radiological examinations to rule out other causes of scoliosis and to ascertain the diagnosis of AIS. The subject exclusion criteria were scoliosis of congenital, neuromuscular, or metabolic etiology, skeletal dysplasia, known endocrine and connective tissue abnormalities, and mental retardation. In the control group, each of the six age- and gender- matched subjects with no history of spinal disease had a straight spine and a normal forward bending test on the physical examination. They were confirmed to be free of any associated medical diseases, spinal deformities, or neurological problems when entered to the study. The study was approved by the Ethics Committee of Chinese Academy of Medical Sciences and Peking Union Medical College Hospital. Written informed consent was obtained from all subjects and their parents before entering the study.

### Isolation and Culture of Cells from Human Bone Marrow

Mononuclear cells were separated by Ficoll gradient centrifugation (density 1.077 g/cm^3^) and depleted of hematopoietic cells using MACS CD45, GlyA, and CD34 micromagnetic beads (Miltenyi Biotec, Inc., Auburn, CA, USA) [33], [34]. The cells were washed twice and plated in T-75 tissue culture flasks at a density of 1×10^6^/ml. Expansion medium contained 57% DMEM/F-12 (Gibco Life Technologies, Paisley, UK), 40% MCDB-201 (Sigma, St.Louis, MO, USA), 2% fetal calf serum (FCS; Gibco), 1× insulin transferrin selenium (ITS; Gibco), 10^−8^ M dexamethasone (Sigma), 10^−4^ M ascorbic acid 2-phosphate (Sigma), 10 ng/ml epidermal growth factor (EGF; Sigma), 10 ng/ml platelet-derived growth factor BB (PDGF-BB; Sigma), 100 U/ml penicillin, and 100 µg/ml streptomycin (Gibco). Once adherent cells were more than 70% confluent, they were detached with 0.125% trypsin and 0.01% EDTA, and replated at a 1∶3 dilution under the same culture conditions. Confluent cells (approximately 2×10^6^) at the third passage were used for the experiments.

### Immunophenotype Analysis

For immunophenotype analysis of BM-MSCs, the cells were detached and washed with phosphate buffered saline (PBS) containing 0.5% bovine serum albumin (BSA; Sigma), and incubated with primary antibodies for 30 min at 4°C. Working concentrations for primary antibodies against human CD29, CD31, CD34, CD44, CD45, CD73, and CD105, (BD Biosciences) were 10 ng/ml. We used same-species, same-isotype irrelevant antibody as the negative control. After washing with PBS containing 0.5% BSA, the cells were incubated with fluorescein isothiocyanate (FITC)-conjugated secondary antibodies for 30 min at 4°C. After three washes, cells were resuspended in PBS and analyzed by flow cytometry with a FACSCalibur flow cytometer. Each measurement contained 10000 events.

### Osteogenic and Adipogenic Differentiation

To identify the MSC capacity for multilineage differentiation, MSCs were cultured under differentiation conditions.

The culture-expanded cells at a density of 2×10^4^/cm^2^ were induced in the following osteogenic medium for 2–3 weeks: Dulbecco's modified Eagle's medium (DMEM) supplemented with 10% FCS, 10 mmol/L β-glycerophosphate, 10^−8^ mol/L dexamethasone, and 0.2 mmol/L ascorbic acid (all from Sigma). Cells were then stained with the alkaline phosphatase (ALP) staining kit (Beyotime, China) to reveal osteogenic differentiation.

To test the adipogenic differentiation ability, the culture-expanded cells at a density of 2×10^4^/cm^2^ were induced for 3 weeks in DMEM supplemented with 10% FCS, 0.5 µmol/L hydrocortisone, 0.5 µmmol/L isobutylmethylxanthine, and 50 µg/ml indomethacin (all from Sigma). At the end of the culture, the cells were fixed in 10% formalin for 10 min and stained with fresh Oil red-O solution (Sigma) to show lipid droplets in induced cells.

### Protein Sample Preparation

Cell pellets were dissolved in lysis buffer (7 M urea, 2 M thiourea, 4% CHAPS, 10 mM Tris). For improved cell lysis, the solution was sonicated on ice for 1 min with 1 s pulse-on and 1 s pulse-off to prevent overheating. After incubation for 30 min at room temperature with repeated vortexing, the samples were centrifuged at 40000×g for 60 min at 10°C to remove unbroken cells from the homogenate. The supernatant was stored in aliquots at −80°C. Protein concentration was determined with the Bradford assay kit (BioRad) using albumin diluted in lysis buffer as the standard.

### DIGE Labeling and Two-Dimensional(2D) Electrophoresis

Cell lysates were labeled with Cy2, Cy3, and Cy5 following the protocols described in the Ettan DIGE User Manual (18-1164-40 Edition AA, GE Healthcare). The DIGE experimental design is shown in Table 1. Typically, 50 µg of lysates (25 µg×2) were labeled with 400 pmol of Cy3 or Cy5, while the same amount of the pool standard containing equal amounts of all samples were labeled with Cy2. Labeling was carried out in the dark on ice for 30 min. Reactions were then quenched by the addition of 1 µL of 10 mM lysine for 10 min on ice. After labeling and quenching, differentially labeled samples were mixed with the pooled Cy2-labeled extracts, and an equal volume of 2× sample buffer (7 M urea, 2 M thiourea, 4% CHAPS, 2% Bio-Lyte, pH 3–10 nonlinear, 2% dithiothreitol) was added to the sample, then the total volume was made up to 450 µl with rehydration buffer (7 M urea, 2 M thiourea, 4% CHAPS, 20 mM DTT, 1% Biolyte).

**Table 1:** DIGE experimental design for samples from AIS group and control group.^a^

Gel	Cy2	Cy3	Cy5
01	Pool of (A 1–6+C1–6) (50 µg)	C1+C2(25 µg each)	A1+A2^b^(25 µg each)
02	Pool of (A 1–6+C1–6) (50 µg)	A3+A4(25 µg each)	C3+C4(25 µg each)
03	Pool of (A 1–6+C1–6) (50 µg)	C5+C6(25 µg each)	A5+A6(25 µg each)

a ) A total of 150 µg of labeled proteins were loaded on each gel for 2D electrophoresis.

b ) 6 samples from AIS group and 6 samples from control group were randomly numbered as A1∼A6 and C1∼C6, respectively.

Two dimensional electrophoresis (2-DE) was performed as previously described with some modifications [35]. IPG strips (24 cm, pH 3–10 nonlinear, GE) and Ettan IPGphor System (GE Healthcare) were used for the first-dimension IEF under the following conditions: 30 V, rapid, 6 h; 60 V, rapid, 6 h; 200 V, rapid, 1 h; 500 V, rapid, 1 h; 1000 V, 1 h, 1000–10000 V, linear, 3 h; and 10000 V, rapid, 60000 Vh. The strips were then treated with a two-step reduction and alkylation step prior to the second-dimension SDS-PAGE. After equilibration with a solution containing 6 M urea, 2% sodium dodecyl sulfate (SDS), 30% glycerol, 50 mM Tris-Cl (pH 8.8), and 0.5% w/v dithiothreitol (DTT) for 15 min at room temperature, the IPG strips were treated with the same solution containing 4.5% w/v iodoacetamide instead of DTT for another 15-min incubation at room temperature. For the second-dimension SDS-PAGE, the strips were overlaid onto 12% polyacrylamide gels (20×24 cm), immobilized to a low-fluorescent glass plate and electrophoresed for 1 h at 10 mA and then for approximately 15 h at 20 mA per gel using an Ettan DALT Six (GE Healthcare). After 2-DE, gels were scanned on the Typhoon 9410 scanner (GE Healthcare) with Ettan DALT gel alignment guides using excitation/emission wavelengths specific for Cy2 (488/520 nm), Cy3 (532/580 nm), and Cy5 (633/670 nm). The intensity was adjusted to ensure that the maximum volume of each image was between 60,000 and 90,000.

### Image Acquisition and Analysis

DeCyder v.6.5 was used to analyze the DIGE images as described in the Ettan DIGE User Manual (GE Healthcare). A DeCyder differential in-gel analysis (DIA) module was performed for image analysis between samples within the same gel, while a DeCyder biological variation analysis (BVA) module was performed for pairwise image analysis among multiple gels. Briefly, in DIA, the Cy2, Cy3, and Cy5 images for each gel were merged, spot boundaries were automatically detected, and normalized spot volumes ( protein abundance) were calculated. The resulting spot maps were exported to BVA. The best internal standard image was assigned as the “Master,” which was used as a template. The protein spots on the remaining internal standard images were all matched to the master gel to ensure that the same protein spots were compared between gels. Matching of the protein spots across all gels was performed after several rounds of extensive land marking and automatic matching. The match was then checked manually to ascertain the accuracy of the match process. Dividing each Cy3 or Cy5 spot volume with the corresponding Cy2 (internal standard) spot volume within each gel gave a standard abundance, thereby correcting integral variations.

The Student's *t* test was used for statistical analysis of the data. Protein spots that were differentially expressed between the AIS and control groups (|ratio|≥1.3, *p*≤0.05) were marked. Only spots altered consistently in all gels were selected for identification.

### Spot Excision and In-gel Tryptic Digestion

Separate preparative gels were run to obtain sufficient amounts of protein for MS analysis. These gels were fixed and stained with colloidal Coomassie Brilliant Blue (cCBB; Amresco) [36]. Proteins of interest, as defined by the 2D-DIGE/DeCyder analysis, were excised from the cCBB-stained gels for a modified in-gel tryptic digestion procedure. Gel pieces were first destained with 50% ACN and 25 mM of ammonium bicarbonate. Following vacuum drying, the gel pieces were incubated with sequencing-grade modified trypsin (Promega) at a final concentration of 0.01 mg/mL in 25 mM of ammonium bicarbonate (Fluka) or 16 h at 37°C. Then the tryptic peptides were extracted from the gels with 5% TFA at 40°C. After extraction for 30 min, the gels underwent sonication for 3 min with an ultrasonic processor and then another 30 minutes' extraction. After collecting the supernatants, the gels were treated with 2.5% TFA, 50% ACN at 30°C for half an hour, then sonicated for 3 min and subsequently treated with the same solution for another half an hour. The extracts were pooled, vacuum-dried, and redissolved in 0.1% TFA for MS analysis.

### MALDI-TOF/TOF Analysis

A total of 0.8 µl peptides were spotted on to the 384-well stainless steel MALDI target plates and dried, followed by 0.6 µl MALDI matrix (7 mg/mL CHCA and 0.1% TFA and 50% ACN) spotted on the same point. Samples on the MALDI target plates were then analyzed using an ABI 4800 Proteomics Analyzer MALDI-TOF/TOF mass spectrometer (Applied Biosystems). For MS analyses, typically 1000 shots were accumulated for each spot, while for MS/MS analysis, 2000 shots were accumulated. MS/MS analyses were performed using air, at collision energy of 2 kV. MASCOT search engine (version 2.1, Matrix Science) was used to search all of the tandem mass spectra. GPS Explorer™ software version 3.6.2 (Applied Biosystems) was used to create and search files with the MASCOT search for peptide and protein identification. The IPI human database v3.53 (http://www.ebi.ac.uk/IP/IPIhelp.html) was used for the search and was restricted to tryptic peptides. Searches were performed to allow for carbamidomethylation, oxidation, and a maximum of one missed trypsin cleavage. Precursor error tolerance was set to <0.2 Da and MS/MS fragment error tolerance <0.3 Da. The confident identification had a statistically significant (*p*≤0.05) protein score (based on combined mass and mass/mass spectra) and best ion score (based on mass/mass spectra). Proteins that appeared in the database under different names and accession numbers were eliminated. If more than one protein was identified in one spot, the single protein member with the highest protein score (top rank) was singled out from the multiprotein family.

### Western Blot Analysis

Five differentially expressed proteins of the 25 identified proteins, pyruvate kinase M2 (PKM2), annexin A2, heat shock 27 kDa protein (HSP27), γ-actin and β-actin were further validated by Western blot analysis, using ubiquitous housekeeping protein GAPDH as the loading control.

Samples were run on 12% SDS-polyacrylamide gels and transferred onto nitrocellulose (NC; GE) membranes in a trans-blot electrophoresis transfer cell (Bio-Rad). The membranes were blocked for 1 h at room temperature in 20 mM Tris-HCl, 140 mM NaCl, pH 7.5, 0.05% Tween-20 (TBST) containing 5% skim milk. The primary antibodies used were anti-glyceraldehyde-3-phosphate dehydrogenase (GAPDH) mouse monoclonal antibody (diluted 1∶2000, ProteinTech), anti-β-actin mouse monoclonal antibody (diluted 1∶1000, ProteinTech), anti-γ-actin mouse monoclonal antibody (diluted 1∶1000, Sigma), purified anti-annexin A2 mouse monoclonal antibody (diluted 1∶500, BD), anti-HSP27 mouse monoclonal antibody (diluted 1∶1000, Cell Signal Technology), and anti-PKM2 rabbit polyclonal antibody (diluted 1∶1000, Cell Signaling Technology). After washing five times with TBST, 3 min each time, membranes were incubated with each primary antibody at room temperature for 2 h. Blots were then washed for additional five times with TBST and incubated with corresponding rabbit anti-mouse or goat anti-rabbit peroxidase-conjugated secondary antibody (diluted 1∶10000, Zhongshan Goldenbridge Technology) for 1 h at room temperature. After washing five times with TBST, immunoreactive complexes were visualized using ECL reagents (Supersignal West Pico Chemiluminescent Substrate, Thermo). All of the membranes were exposed on the same X-ray film and scanned by Image Scanner (UMAX, Amersham Biosciences). A semiquantitative analysis based on OD was performed by QuantitiOne software (Bio-Rad).

## Results

### The Biological Characteristics of MSCs

**Figure 1:**
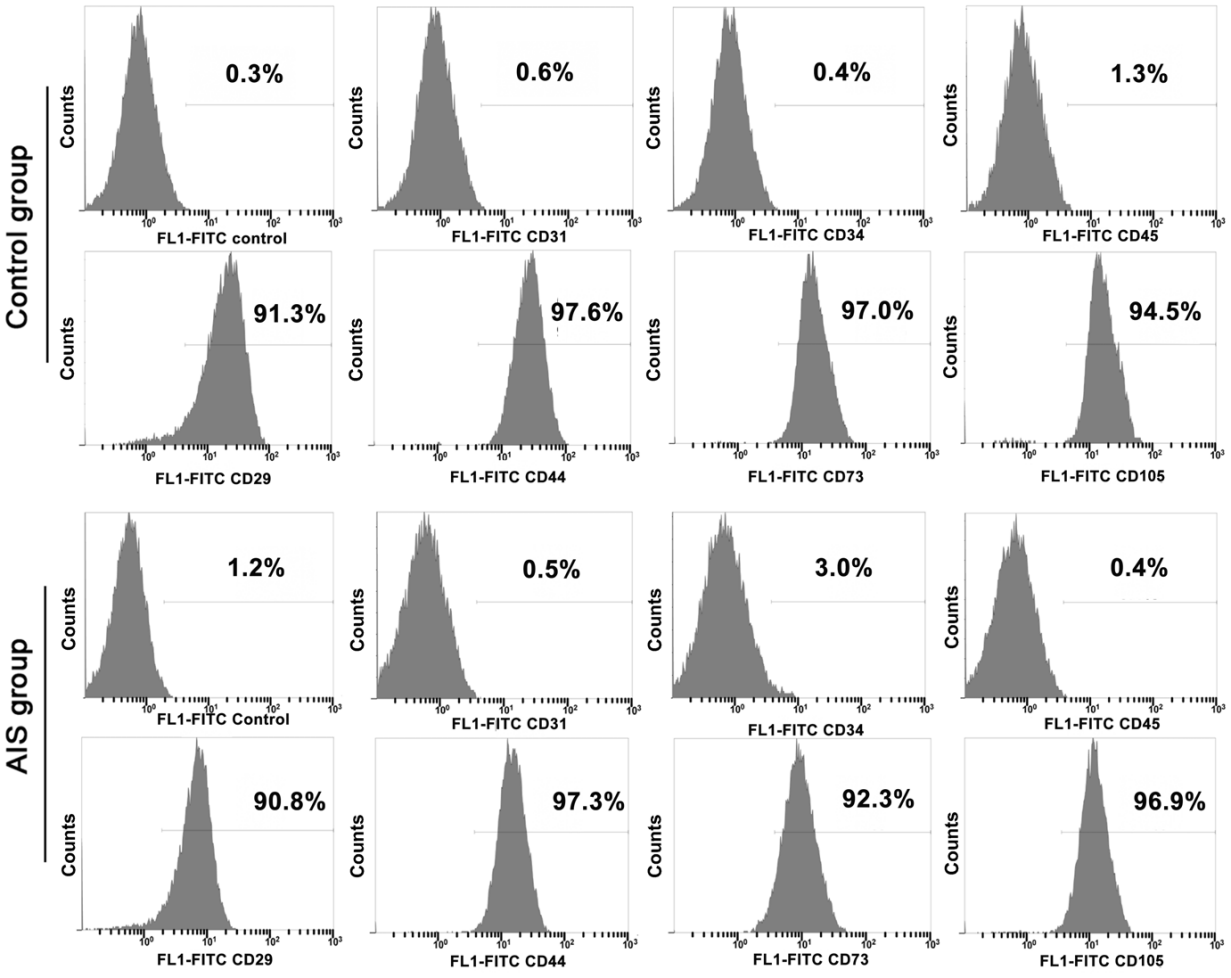
The immunophenotypes of MSCs from control and AIS groups. The figure shows immunophenotypes of MSCs isolated from non-AIS patients with lower-leg fracture (control) and AIS patient detected by FACS.

**Figure 2:**
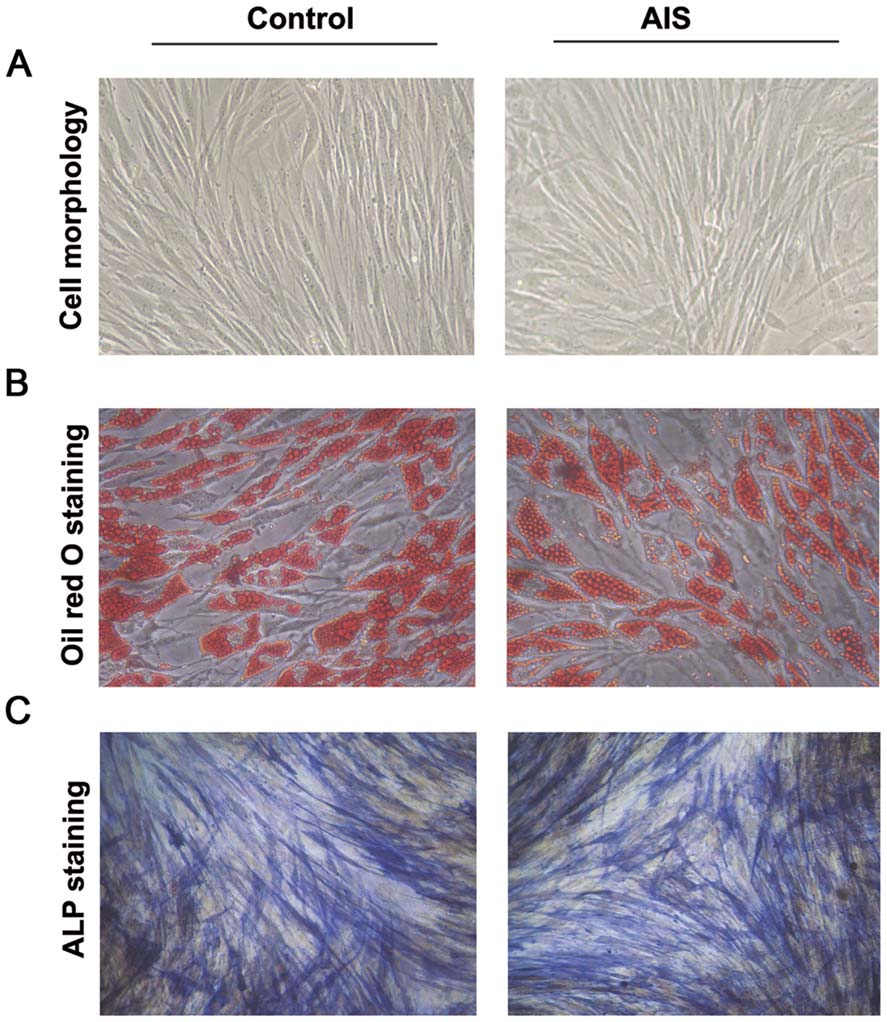
Cell morphology, osteogenic and adipogenic differentiation abilities of MSCs from control and AIS groups. (A) Cell morphology of MSCs of the control and AIS groups at the 3rd passage (Magnification 100×). (B) Oil red O staining of MSCs of the control and AIS groups after adipogenic induction for two weeks (Magnification, 200×). (C) ALP staining of MSCs of the control and AIS groups after osteogenic induction for two weeks (Magnification, 200×).

All of the cultured cells from AIS patients and non-AIS patients with lower-leg fracture grew well and displayed fibroblast-like morphology in culture medium when observed under a light microscope. To demonstrate that these isolated cells were MSCs, we investigated their immunophenotypes coupled with multilineage differentiation capacities. These cells from both the AIS and control groups were persistently negative for CD31, CD34, and CD45, but expressed high levels of CD29, CD44, CD73, and CD105 (Figure 1). Furthermore, they can be differentiated into osteoblasts and adipocytes, which was verified by ALP and Oil Red O staining (Figure 2).

### Identification of Proteins Using 2-DE

We analyzed the MSC proteome of a group of 12 individuals (six AIS patients and six non-AIS controls) by 2D-DIGE. Globally, 1658±37 spots (mean ± SD; n = 6, AIS; n = 6, non-AIS controls) were detected on each analytical gel loaded with 50 µg of total protein lysate per sample type (AIS or control).

**Figure 3:**
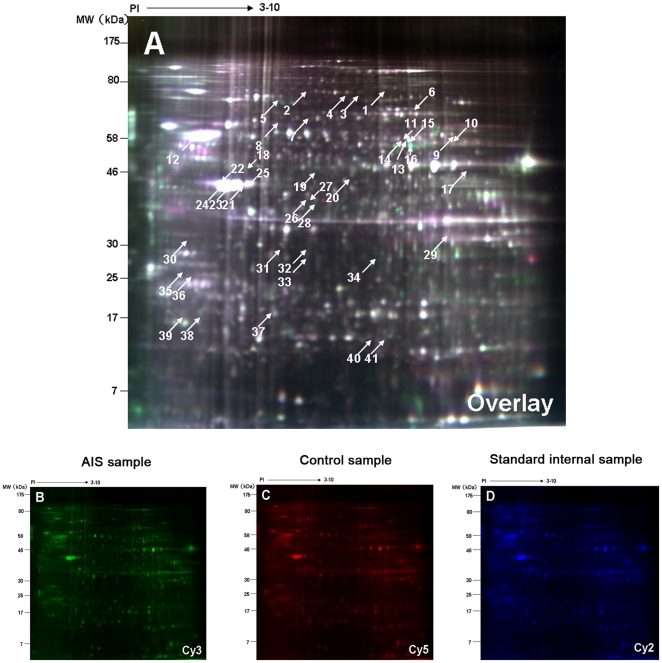
Identification of the differentially expressed proteins using 2-DE. The figure displays Representative 2D-DIGE images of MSCs labeled with Cy3(B) , Cy5(C),Cy2(D) and overlay of the three color images(A) derived from a single gel. Spots for which the quantitative statistical analysis revealed >1.3-fold protein expression change and a Student's t test *p* value less than 0.05 in the AIS group were annotated by numbers according to pI and Mw. The annotated protein spots were cut out of the gel and subjected to tryptic digestion followed by MS analysis.

As for the analyzed gel spots, we selected those fulfilling two criteria: expression change of at least 1.3-fold compared to the control group and a significant t test result (n = 6 in each group, *p*<0.05). The comparison between MSCs from AIS and control groups resulted in the identification of 41 significantly different spots, the locations of which on the 2D gels were labeled with spot ID (Figure 3). These spots were excised manually from silver-stained gels and were identified by MS. A total of 34 spots of the 41 excised (82.9%), representing 25 different gene products, were unambiguously identified as either up-regulated or down-regulated. The mass spectra of five significantly differential proteins related to bone metabolism, including PKM2, annexin A2, , β-actin, γ-actin, and HSP27, are presented in Figure S1, S2, S3, S4, S5, respectively.

Table 2 provides a list of identified proteins with their respective spot ID, protein name, IPI ID, the theoretical pI/MW, the percentage of sequence coverage, p-value, total protein score, best ion score, and peptide sequence determined by MS. Five of these 25 identified proteins were found in more than one spot, according to similar molecular masses and pIs: UDP-glucose dehydrogenase (IPI 00031420) was found in spots 9 and 10; ATP synthase subunit α, mitochondrial (IPI 00440493) in spots 13, 14, and 16; γ-actin (IPI 00021440) in spots 21, 22, 23, 24, and 25; β-actin (IPI 00894365) in spots 26 and 27, and annexin A2 (IPI 00455315) in spots 29 and 32.

**Table 2:** Differentially expressed proteins identified in MSCs from AIS group and Control group by 2-DE and MS or MS/MSa).

Spot No.b)	Protein descriptionc)	IPI Nod)	MWe)	PIf)	Sequence coverageg)	P value	Average Ratioh)	Total protein Scorei)	No.of matched peptide (MS)j)	Peptides sequencek)
3	Moesin	IPI00872814	67644.8	6.09	25%	0.026	−1.4	99	16	APDFVFYAPR(48)
4	Glycyl-tRNA synthetase	IPI00783097	83086.6	6.61	18%	0.006	−1.35	131	12	LPFAAAQIGNSFR(50) TLYVEEVVPNVIEPSFGL(43)
5	Heat shock 70 kDa protein 9	IPI00007765	73634.8	5.87	25%	0.026	−1.51	258	14	LLGQFTLIGIPPAPR(49) AQFEGIVTDLIR(43) VQQTVQDLFGR(40)
6	WD repeat-containing protein 1	IPI00746165	66151.9	6.17	23%	0.0082	−1.34	97	15	VFASLPQVER(28)
7	FK506 binding protein 10	IPI00334818	51992.1	5.23	22%	0.0076	1.31	114	11	NTLVAIVVGVGR(38)
8	T-complex protein 1 subunit alpha	IPI00290566	60305.6	5.8	20%	0.012	−1.34	140	10	EQLAIAEFAR(38)
9	UDP-glucose dehydrogenase	IPI00031420	54989.2	6.73	42%	0.0039	−1.44	238	13	RIPYAPSGEIPK(45) VLIGGDETPEGQR(41) LAANAFLAQR(38)
10	UDP-glucose dehydrogenase	IPI00031420	54989.2	6.73	35%	0.02	−1.39	234	15	EQIVVDLSHPGVSEDDQVSR(56) LAANAFLAQR(47)
11	Glucose-6-phosp-hate 1-dehydrogenase	IPI00289800	59219	6.39	26%	0.0029	1.34	288	17	LSNHISSLFR(48) IIVEKPFGR(38)
12	Tubulin, beta	IPI00645452	47736	4.7	25%	0.019	1.49	235	11	FPGQLNADLR(56) YLTVAAVFR(48) GHYTEGAELVDSVLDVVR(41)
13	ATP synthase subunit alpha, mitochondrial	IPI00440493	59713.6	9.16	26%	1.00E-05	1.35	170	11	TGAIVDVPVGEELLGR(66) EAYPGDVFYLHSR(47)
14	ATP synthase subunit alpha, mitochondrial	IPI00440493	59713.6	9.16	31%	0.046	1.77	190	13	TGAIVDVPVGEELLGR(53) EAYPGDVFYLHSR(50)
15	Pyruvate kinase M2	IPI00847989	49865.9	7.96	41%	0.042	2.81	185	10	LDIDSPPITAR(42)
16	ATP synthase subunit alpha, mitochondrial	IPI00440493	59713.6	9.16	26%	0.0048	1.56	183	11	TGAIVDVPVGEELLGR(61) EAYPGDVFYLHSR(51)
17	Alpha-enolase	IPI00465248	47139.3	7.01	35%	0.0033	−1.3	264	12	VVIGMDVAASEFFR(64) AAVPSGASTGIYEALELR(59) VVIGMDVAASEFFR(41)
18	Cytochrome b-c1 complex subunit 1, mitochondrial	IPI00013847	52612.4	5.94	27%	0.014	1.34	227	10	DVVFNYLHATAFQGTPLAQAVEGPSENVR(102) IAEVDASVVR(59)
19	Serpin H1	IPI00032140	46411.2	8.75	28%	0.021	1.51	188	10	LYGPSSVSFADDFVR(71) DTQSGSLLFIGR(54)
20	Ornithine aminotransferase, mitochondrial	IPI00022334	48504.2	6.57	32%	0.038	1.32	206	13	HQVLFIADEIQTGLAR(50) FAPPLVIKEDELR(48)
21	γ-actin	IPI00021440	41765.8	5.31	51%	0.015	−2.02	263	15	SYELPDGQVITIGNER(54) QEYDESGPSIVHR(45)
22	γ-actin	IPI00021440	41765.8	5.31	44%	0.016	−1.82	411	12	DLYANTVLSGGTTMYPGIADR(118) SYELPDGQVITIGNER(92) IWHHTFYNELR(59) AVFPSIVGRPR(41)
23	γ-actin	IPI00021440	41765.8	5.31	50%	0.014	−1.45	482	14	DLYANTVLSGGTTMYPGIADR(104) SYELPDGQVITIGNER(79) TTGIVMDSGDGVTHTVPIYEGYALPHAILR(63) IWHHTFYNELR(56) AGFAGDDAPR(49)
24	γ-actin	IPI00021440	41765.8	5.31	46%	0.0068	−1.34	403	13	DLYANTVLSGGTTMYPGIADR(129) SYELPDGQVITIGNER(95) IWHHTFYNELR(55) AVFPSIVGRPR(39)
25	γ-actin	IPI00021440	41765.8	5.31	48%	0.027	−1.63	339	14	SYELPDGQVITIGNER(82) DLYANTVLSGGTTMYPGI(77) IWHHTFYNELR(53)
26	β-actin	IPI00894365	39200.5	5.4	24%	0.013	−1.46	88	7	SYELPDGQVITIGNER(43)
27	β-actin	IPI00894365	39200.5	5.4	27%	0.017	−1.4	70	8	SYELPDGQVITIGNER(35)
28	Macrophage-capping protein	IPI00027341	38493.5	5.88	16%	0.0043	1.51	71	4	QAALQVAEGFISR(57)
29	Annexin A2	IPI00455315	38579.8	7.57	35%	0.038	−1.38	80	8	GVDEVTIVNILTNR(46)
30	Elongation factor 1-delta	IPI00023048	31102.8	4.9	39%	0.022	−1.38	284	10	SLAGSSGPGASSGTSGDHGELVVR(79) IASLEVENQSLR(68)
31	Inorganic pyrophosphatase	IPI00015018	32639.2	5.54	32%	0.041	−1.34	140	8	VIAINVDDPDAANYNDINDVKR(68)
32	Annexin A2	IPI00455315	38579.8	7.57	33%	0.03	−1.47	103	10	GVDEVTIVNILTNR(62)
36	Heterogeneous nuclear ribonucleoprotein K	IPI00514561	47527.6	5.46	28%	0.024	−1.31	135	9	LLIHQSLAGGIIGVK(66)
37	Heat shock 27 kDa protein	IPI00025512	22768.5	5.98	36%	0.022	−1.52	163	8	LFDQAFGLPR(61) LATQSNEITIPVTFESR(40)
39	Glyoxalase I	IPI00220766	20764.2	5.12	27%	0.033	1.4	121	7	RFEELGVK(37)
40	Proteasome subunit, beta type	IPI00789577	12074.3	9.86	57%	0.0062	−1.38	68	5	LYIGLAGLATDVQTVAQR(39)

a) Spots for which the volume ratio was ≥1.3 based on DeCyder software analysis were identified by MALDI-TOF/TOF MS.

b) Spots referring to Figure 3.

c) Protein description: name of each matched protein in IPI human database v3.53 (http://www.ebi.ac.uk/IP/IPIhelp.html) by data searching.

d) IPI No: Protein ID accessed from IPI human database v3.53.

e) MW: theoretical molecular weight of the matched protein in Da.

f) PI: theoretical isoelectric point of the matched protein.

g) Percent of identified sequence to the complete sequence of the known protein.

h) Average volume ratio in AIS group compared to control group.

i) Total protein score based on combined mass and mass/mass spectra.

j) No.of matched peptides: the number of peptides (MS) matched to the candidate protein.

k) All the spots had high-probability results by MASCOT search, and there was at least one peptide analyzed by MS/MS in each spot. Parts of the sequence, determined by MS/MS, indisputably confirm the peptide.

### Functional Classification and Subcellular Location of Identified Proteins

**Figure 4:**
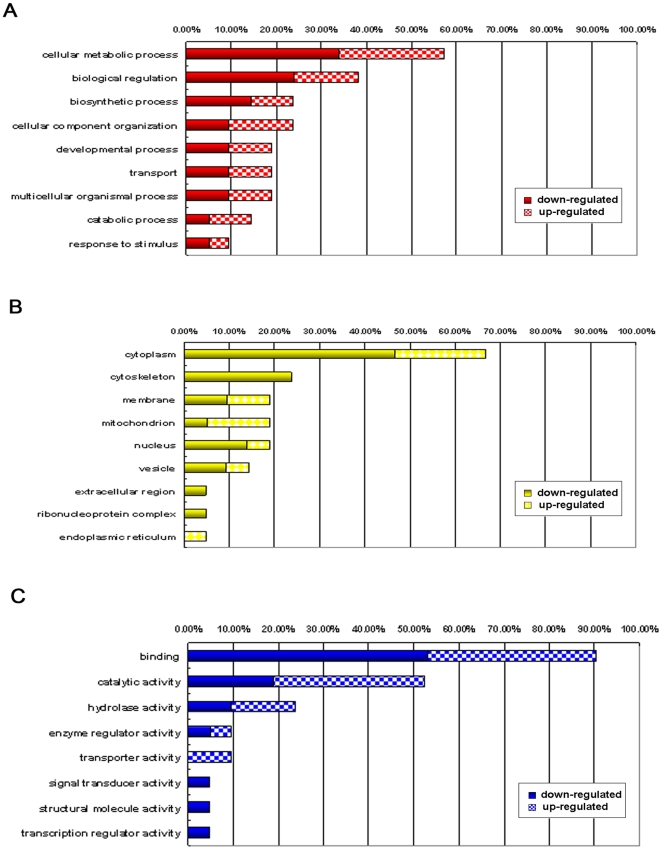
Functional classification and subcellular location of identified proteins. Distribution of the identified proteins are presented according to their (A) biological processes, (B) cellular component, and (C) molecular function. Assignments were made using the GOFACT program (http://61.50.138.118/gofact) based on Gene Ontology (GO) terms. Prior to the analysis, the differentially regulated proteins were listed as up-regulated or down-regulated. For example: 57% of the total 34 proteins (or 25 gene products), including 33.3% down-regulated proteins and 23.8% up-regulated proteins, were involved in the cellular metabolic process.

The proteins listed in Table 2 were classified into different groups according to their biological process, molecular function, and cellular component using the GOFACT program (http://61.50.138.118/gofact) based on Gene Ontology (GO) terms [37]. The majority of proteins that changed at least 1.3-fold (with *p*<0.05) were involved in cellular metabolic (57.1%), biological regulation (38.1%), and biosynthetic processes (23.8%) (Figure 4A). Furthermore, an analysis of subcellular distribution of the differential proteins allowed the differentiation of 9 different categories (Figure 4B). The majority of proteins were located in the following five subcellular positions: cytoplasm (66.7%), cytoskeleton (23.8%), membrane (19%), mitochondrion (19%), and nucleus (19%), while the remaining reside in the vesicle, extracellular region, ribonucleoprotein complex, and endoplasmic reticulum. Interestingly, five of twenty-five differentially expressed proteins (β-actin, γ-actin, HSP27, WD repeat-containing protein 1, and moesin) were localized in the cytoskeleton, all of which were down-regulated in our experiment. Additionally, the classification of the gene products based on their molecular function resulted in 8 different categories (Figure 4C), among which the binding function represents the largest group (90.5%). The proteins were classified into the following categories: binding, catalytic activity, hydrolase activity, enzyme regulator activity, transport, signal transducer activity, structural molecule activity, and transcription regulator activity.

### Protein Validation by Western Blot

**Figure 5:**
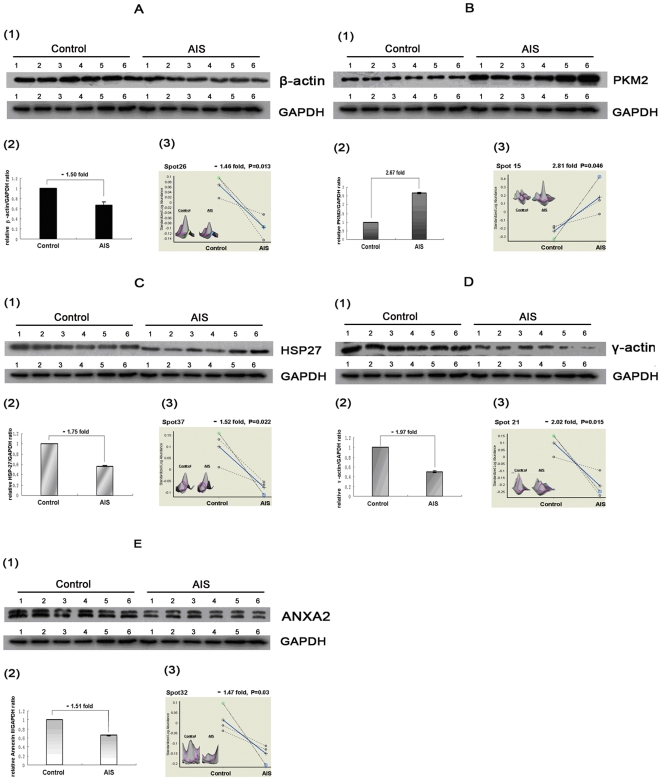
Validation of five differentially expressed proteins. The figure shows validation of the differential expression of (A) β-actin, (B) PKM2, (C) HSP27, (D) γ-actin, and (E) annexin A2 (ANXA2) by western blot analysis and comparative data (with fold changes and Student's t test values) for their corresponding filtered spots by Decyder software. The expression patterns of these proteins show good correlation. Control = control group; AIS = AIS group. 1) Representative 1-D Western blot analysis of above proteins (all performed on 6 AIS samples and 6 control samples), GAPDH was used as the internal control. 2) Densitometric analysis of bands from western blot images by ImageQuant software. Values were presented as relative ratio of differentially expressed protein/GAPDH(y-axis) in MSCs from the control and AIS groups (x-axis), normalized to 1 in control group, n = 6, *p*<0.05, Student's t test. Fold changes of the ratio in the AIS group compared to the control group are also presented. 3) Graphic views show the standardized log abundance of spot volume (y-axis) against the changes of proteins between the AIS and Control groups (x-axis) in all three gels (with fold changes and Student's t test values). DeCyder Software 3D view of these 5 differentially expressed protein spots is also shown.

Significantly differential proteins that were most likely to have biological importance, including PKM2, annexin A2, HSP27, γ-actin, and β-actin, were detected by Western blot analysis for their expression. The results are displayed in Figure 5. The expression of β-actin, HSP27, γ-actin, and annexin A2 was down-regulated, and that of PKM2 was up-regulated in the AIS group. All of these change patterns of the selected proteins were in agreement with 2-DE results. Therefore, the results of Western blot analysis confirmed the reliability of the proteomic analysis.

## Discussion

The etiology and pathophysiologic process underlying AIS remains unclear despite the number of studies performed. Current views maintain that AIS is a multifactorial disease involving genetic [10], [38], skeletal [39], [40], environmental [41]–[43], biochemical [13], and neurohormonal factors [14]–[16].

It is generally recognized that abnormal growth is associated with the development and progression of the scoliotic curves [44]–[45]. Furthermore, persistent general osteopenia of AIS patients indicates imbalance between bone resorption and bone formation in AIS [19]–[25]. Interestingly, recent findings [26], [32] of diminished numbers of osteoblast and lower osteogenic differentiation abilities of MSCs from AIS patients rendered the MSCs impairment one possible mechanism of osteopenia in AIS, which has aroused growing concern.

The findings of abnormal bone growth and development in AIS, together with functional characteristics of MSCs, strongly suggest that MSCs may play a significant role in the etiology and pathogenesis of AIS. However, due to the lack of research in this area, we know very little of the biological characteristics, proteomic alterations, and the possible role of MSCs in the pathogenesis of AIS and accompanying generalized osteopenia. Therefore, to investigate the molecular mechanism of decreased osteogenic differentiation ability of MSCs and gain an insight into the pathogenesis of AIS, we employed 2D-DIGE and MS-based proteomic approaches to explore the differential protein expression patterns in MSCs of AIS and non-AIS controls. To the best of our knowledge, this study is the first research on AIS in the field of proteomics, and also one of only a few studies focused on MSCs from AIS patients.

As previously described, 41 spots were revealed with at least 1.3-fold changes in expression and 25 differentially expressed proteins were successfully identified by MALDI-TOF/TOF-MS. Due to their significant expression alterations as well as potential functional relevance to bone growth and development, five of these proteins, including PKM2, annexin A2, HSP27, γ-actin, and β-actin, were chosen to be further validated by Western blot analysis. These five differential proteins will be discussed in the following section.

### PKM2

It is well known that different isoenzymes of pyruvate kinase are expressed depending on the metabolic responsibilities of the various cells and tissues. Among them, pyruvate kinase isoenzyme type M2 (PKM2, M2-PK) is characteristic of cells with high rates of nucleic acid synthesis, including most of the proliferating cells, such as adult stem cells, embryonic cells, and tumor cells [46], [47].

According to previous reports, PKM2 plays an important role in both cell proliferation and differentiation. In tumor cells, PKM2 has been regarded as an important metabolic sensor to adapt tumor metabolism to varying nutrient and oxygen supply conditions, thus facilitating cell proliferation and survival [48]. It was also observed in the BB13 cell line that PKM2 translocated to the nucleus by IL-3 enhanced EGF-induced proliferation [49]. In addition, using NIH3T3 cell line, Gilles A's finding suggested that PKM2 could regulate cell proliferation, cell growth and apoptosis in a glucose supply-dependent manner [50].

More importantly, inhibition of PKM2 induces a significant decrease in the population doubling (PDL) and cell proliferation rates, as well as an increase in cell size [50]. Contrary to this, overexpression of PKM2 was found to enhance cell proliferation in the absence of interleukin-3 [49]. All of these studies suggest that PKM2 plays an important role in cell proliferation.

Furthermore, it has been reported that PKM2 is able to stimulate Oct-4-mediated transcriptional activation. Oct-4, as a central mediator of the undifferentiated pluripotent state of embryonic stem cells, may prevent expression of genes activated during differentiation [51]. These observations indicate that PKM2 could negatively regulate cell differentiation through modulating the transactivation potential of the Oct-4 transcription factor.

In our study, PKM2 was significantly up-regulated in the MSCs of AIS, suggesting that the proliferation ability of MSCs in AIS might be increased. In contrast to our speculation, Park et al found the proliferation rate of MSCs obtained from AIS patients to be similar to that of the control subjects [32]. However, the author also states that the number of patients included in their study was relatively small and suggests performing larger studies. Therefore, the exact alteration of MSC proliferation ability in AIS needs further large-scale studies to clarify.

Additionally, due to the negative correlation between PKM2 and cell differentiation, up-regulation of PKM2 in the AIS-MSCs in our experiment indicates decreased differentiation ability of MSCs in AIS, which not only support previous observation of reduction of osteogenic differentiation ability of AIS-MSCs, but also provide a possible mechanism of persistent general osteopenia of AIS patients.

### Annexin A2

In this study, annexin A2 was differentially down-regulated in MSCs obtained from AIS patients. These molecules belong to the annexin protein family, which have in common that they bind to acidic phospholipids in the presence of calcium [52]–[55]. Annexin A2 contains four 70–80 amino acid repeats with an annexin consensus sequence. These four repeats form the conserved core region, which is responsible for the Ca^2+^-dependent binding of the proteins to phospholipids [56].

Annexin A2, which is highly expressed by osteoblasts [57], osteoarthritic chondrocytes [58], [59], hypertrophic and terminally differentiated growth plate chondrocytes [60], has been demonstrated to play a significant role in both intramembrane and intrachondral ossification.

In growth plate chondrocytes, it has been found that annexin A2 is essential to form Ca^2+^ channels in both the plasma membrane [56] and matrix vesicles [61], which are particles released from the plasma membrane of mineralizing cells, and thereby initiating the mineralization process. The annexin-mediated alteration in Ca^2+^ homeostasis thereby regulates a whole sequence of events eventually leading to matrix mineralization. Furthermore, it has also been reported that inhibiting annexin channel function prevents terminal differentiation and the mineralization of growth plate chondrocytes *in vitro* [60].

Similarly, human osteoarthritic chondrocytes also release annexin A2-containing matrix vesicles, which initiate mineral formation [57]. Annexin A2, which is not detectable in the upper, middle, and deep zones of healthy human articular cartilage, is expressed by chondrocytes in the upper zone of early- and late-staged human osteoarthritic cartilage [57].

Annexin A2 has also been shown to play an important role in the mineralization of osteoblastic cells. It was found that overexpression of annexin A2 led to increased ALP activity, which would be further elevated following differentiation [62]. Since ALP has been considered a valuable indicator for bone development and differentiation, annexin A2 may alter ALP activity, thereby facilitating mineralization, a terminal step in the differentiation process of osteoblastic cells.

In studies of other cell types, there is much evidence to support the association between annexin A2 and cellular differentiation. For instance, annexin A2 expression is affected when myeloid cell lines are induced to differentiate by stimulation with all-trans-retinoic acid (ATRA) [63]. As discussed above, annexin A2 may therefore be an important player in cellular differentiation and related disorders.

In this study, we demonstrated that annexin A2 was down-regulated significantly in MSCs from AIS patients, which was consistent with the previously described decrease in osteogenic differentiation ability. It is likely that annexin A2 plays an important role in osteogenic differentiation of MSCs from AIS patients and exerts further influence on both intramembrane and endochondral ossification in AIS. Therefore, annexin A2 might be responsible, at least in part, for the low bone mass in AIS, although the exact mechanism in etiology of AIS needs further research.

### HSP27

The mammalian small stress protein HSP27 (also denoted HSP28 and in murine cells, HSP25) belongs to the HSP family whose synthesis is induced or stimulated by heat shock and other forms of stress [64]. We would like to discuss HSP27 from the following two aspects.

#### (1) HSP27 and Cell Differentiation

Recent studies have revealed that expression of the small heat shock (or stress) proteins (sHSP), including that of HSP27, is closely linked to changes in the state of cell differentiation. During the process of endochondral bone formation, sHSPs are differentially expressed in a stage-specific manner [65]. Similar expression of sHSPs was observed during the differentiation of various mammalian cell types, such as embryonal carcinoma, embryonic stem cells [66], mouse Ehrlich ascites tumor cells [67], [68], normal B cells, B lymphoma [69], osteoblasts, promyelocytic leukemia cells [70] and NB4 promyelocytic cells [71].

In addition, transient accumulation of HSP27 has been observed during phorbol ester-induced monocytic differentiation of human HL-60 cells [72], as well as during ATRA-induced granulocytic differentiation of these cells [73].

In this study, HSP27 was down-regulated in MSCs from AIS patients. Together with the findings of previous studies, HSP27, as a mediator of cell differentiation, might be related to decreased differential ability of MSCs and clinical osteopenia in AIS patients.

#### (2) HSP27, HSP70 and Enviornmental Susceptibility

Many etiological studies of AIS suggest that idiopathic scoliosis is a genetic trait modified by environmental factors. Van Rhijn et al [74] stated that differences in the development and progression of scoliosis, as well as age at detection (juvenile vs. adolescent) may be caused by environmental influences. In another study, van Rhijn et al [41] noted that only half of twin pairs showed a difference in lateral Cobb angles of less than 10°, suggesting that curve severity may be affected by environment. In addition, Hermus [43] reported a monozygotic twin pair that was described to be concordant for idiopathic scoliosis, but with different apical levels, magnitudes, and age at detection, further stressing the importance of environmental factors. However, the molecular mechanism of environmental susceptibility in AIS patients remains unclear.

Both HSP27 and HSP70 have been demonstrated as essential to ensure proper folding and intracellular localization of newly synthesized polypeptides [75], [76]. The expression of HSPs (including HSP27 and HSP70), which is activated by unfolded proteins, can enhance the cell's capacity not only to prevent protein aggregation and disassociate such aggregates once formed, but also to isolate such polypeptides in inclusions and selectively degrade them [77], [78].

Since HSP27 and HSP70 can promote refolding, solubilization, and degradation of damaged polypeptides, the loss of this protective response should deteriorate the cell's capacity to handle the mutant or damaged proteins. In Parkinson's disease or Alzheimer's disease, for instance, such changes in the cell's proteolytic capacity, and the general increase in unfolded molecules, should further limit the capacity of the cell to deal with mutant proteins or other abnormal polypeptides and may thus indirectly contribute to the development of these disorders [78]–[80].

Our study showed down-regulation of both HSP27 and HSP70 in MSCs from AIS patients. If these proteins contribute a similar protective factor in AIS as in the diseases we discussed above, then its lower expression might subsequently compromise the capacity of MSCs to cope with aberrant polypeptides or damaged proteins caused by environmental pathogenic factors of AIS.

Taken together, it is tempting to speculate that the loss of these protective mechanisms, at least in part, renders AIS patients more susceptible to environmental factors, and thus eventually leads to the development and progression of scoliosis. The underlying mechanism of HSP27 and HSP70 in the etiology of AIS needs further elucidation.

### β-actin and γ-actin

β-actin is usually found to be constitutively expressed, with the expression values often used for normalization of expression data. Interestingly, in our study, we found significantly decreased β-actin levels in MSCs from AIS patients.

It has been demonstrated that the actin cytoskeleton, consisting of both β-actin and γ-actin, changes from a large number of thin, parallel microfilament bundles extending across the entire cytoplasm in undifferentiated MSCs to a few, thick actin filament bundles located at the outermost periphery in MSC-differentiated osteoblasts [81]. Therefore, it is postulated that the actin cytoskeleton may play a pivotal role in determining the hMSC mechanical properties and modulation of cellular mechanics during stem-cell osteodifferentiation [82].

During osteogenic differentiation, it was also observed that alterations in the cytoskeletal organization affect the expression of osteogenic differentiation markers, including alkaline phosphatase activity and calcium deposition [81]. More interestingly, it was revealed that disrupting actin in hMSCs increased adipogenesis and decreased osteogenesis when compared to untreated controls, suggesting that the actin cytoskeleton might be important in the commitment process [83]. In addition, it was reported that disruption of the actin cytoskeleton blocks osteoblastic differentiation of cells infected with constitutively active RhoA, which is one of the key regulators of cytoskeletal contractility [84].

Both β-actin and γ-actin were down-regulated in MSCs from AIS patients in our study, which was in agreement with previous reports of decreased osteogenic differentiation capacity of MSCs in AIS patients. Therefore, these data suggest that alteration of the actin cytoskeleton might be involved in the pathological mechanism of persistant general osteopenia in AIS. Additionally, it is worth mentioning that all five identified proteins in our experiment that were localized in cytoskeleton (β-actin, γ-actin, HSP27, WD repeat-containing protein 1, and moesin) were differentially down-regulated, indicating that disruption or inhibition of the cytoskeleton might contribute to the development and progression of AIS.

Down-regulation of β-actin, HSP27, γ-actin, and annexin A2, and up-regulation of PKM2, was reported for the first time as associated with the etiology of AIS in this experiment. Furthermore, the expression alterations of these five proteins indicate increased proliferation ability of MSCs and decreased osteogenic differentiation ability in AIS, the latter of which was also consistent with previous studies of AIS-MSC and might be one of mechanisms causing clinical osteopenia of AIS. In addition, the discovery of a total of 25 differentially expressed proteins in AIS patients may provide a valuable basis for further research on the characteristics of AIS-MSCs as well as the abnormal bone growth and development in AIS.

In summary, we have described the differential proteome of BM-MSCs from AIS patients for the first time. Our high-throughput proteomic approach based on 2D-DIGE technology followed by MS analysis has produced the differential proteome profile of BM-MSCs in AIS. A total of 25 proteins were identified as either up-regulated or down-regulated. These proteins might be involved in proliferation, differentiation, and other activities of MSCs. Furthermore, these differentially expressed proteins might play a significant role, in not only the causal mechanism of osteopenia in AIS, but also the AIS initiation and development. The identification of these proteins provides us important informations in understanding the underlying etiological mechanisms of AIS. Further studies are required to clarify the association between the possible changes of MSCs and the pathogenesis of AIS.

## Supporting Information

Figure S1 **The MS and MS/MS spectra of PKM2.** The figure displays the MS spectrum (A) and MS/MS spectrum marked with b ions and y ions (B) for PKM2 identification. The sequence of precursor at *m*/*z*1197.61 was analyzed by MS/MS to be LDIDSPPITAR. This protein was identified to be PKM2 after database searching.
(TIF)

Figure S2 **The MS and MS/MS spectra of annexin A2.** The figure displays the MS spectrum (A) and MS/MS spectrum marked with b ions and y ions (B) for annexin A2 identification. The sequence of precursor at *m*/*z*1542.88 was analyzed by MS/MS to be GVDEVTIVNILTNR. This protein was identified to be annexin A2 after database searching.
(TIF)

Figure S3 **The MS and MS/MS spectra of β-actin.** The figure displays the MS spectrum (A) and MS/MS spectrum marked with b ions and y ions (B) for β-actin identification. The sequence of precursor at m/z1790.97 was analyzed by MS/MS to be SYELPDGQVITIGNER. This protein was identified to be β-actin after database searching.
(TIF)

Figure S4 **The MS and MS/MS spectra of γ-actin.** The figure displays the MS spectrum (A) and two MS/MS spectra marked with b ions and y ions (B, C) for γ-actin identification. The sequences of precursor at m/z1516.66 and m/z1790.82 were analyzed by MS/MS to be QEYDESGPSIVHR and SYELPDGQVITIGNER, respectively. This protein was identified to be γ-actin after database searching.
(TIF)

Figure S5 **The MS and MS/MS spectra of HSP27.** The figure displays the MS spectrum (A) and two MS/MS spectra marked with b ions and y ions (B, C) for HSP27 identification. The sequences of precursor at m/z1163.58 and m/z1905.92 were analyzed by MS/MS to be LFDQAFGLPR and LATQSNEITIPVTFESR, respectively. This protein was identified to be HSP27 after database searching.
(TIF)
