# Supplementary figures and images for: Differential Proteome Analysis of Bone Marrow Mesenchymal Stem Cells from Adolescent Idiopathic Scoliosis Patients
Source: PLoS One. 2011 Apr 22;6(4):e18834. doi: 10.1371/journal.pone.0018834 (PMC3081308; doi:10.1371/journal.pone.0018834)

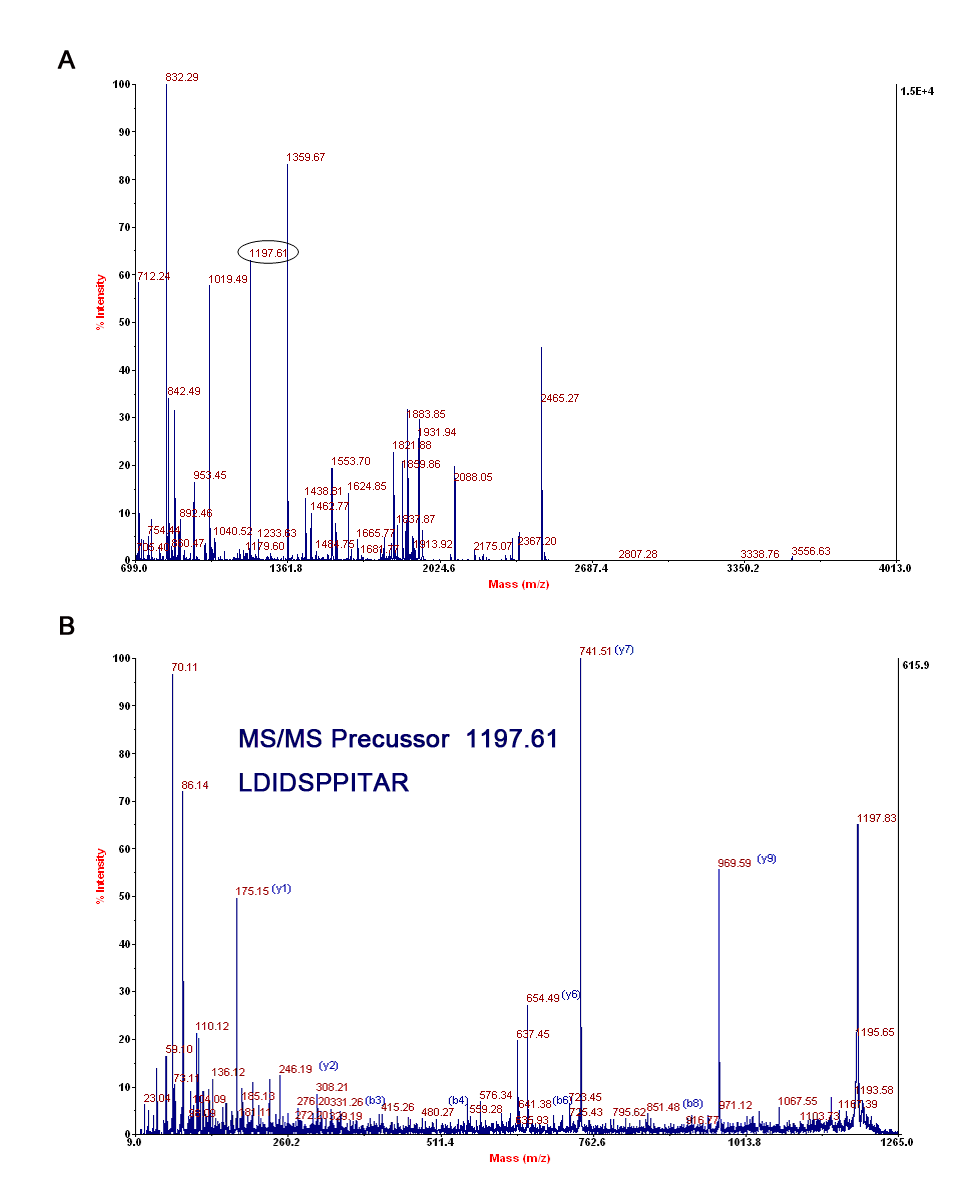

Supplement: Figure S1 — The MS and MS/MS spectra of PKM2. The figure displays the MS spectrum (A) and MS/MS spectrum marked with b ions and y ions (B) for PKM2 identification. The sequence of precursor at m/z1197.61 was analyzed by MS/MS to be LDIDSPPITAR. This protein was identified to be PKM2 after database searching. (TIF) [file pone.0018834.s001.tif]

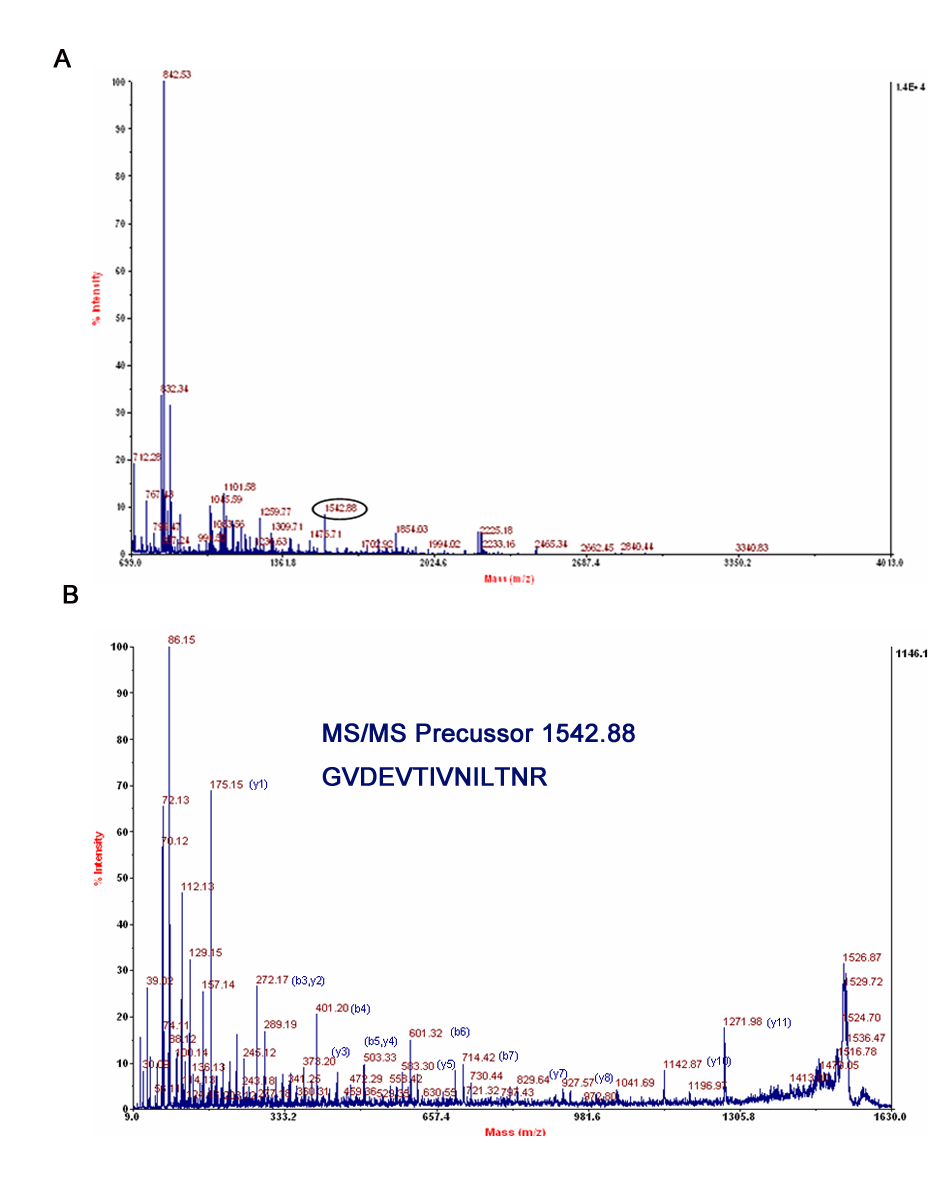

Supplement: Figure S2 — The MS and MS/MS spectra of annexin A2. The figure displays the MS spectrum (A) and MS/MS spectrum marked with b ions and y ions (B) for annexin A2 identification. The sequence of precursor at m/z1542.88 was analyzed by MS/MS to be GVDEVTIVNILTNR. This protein was identified to be annexin A2 after database searching. (TIF) [file pone.0018834.s002.tif]

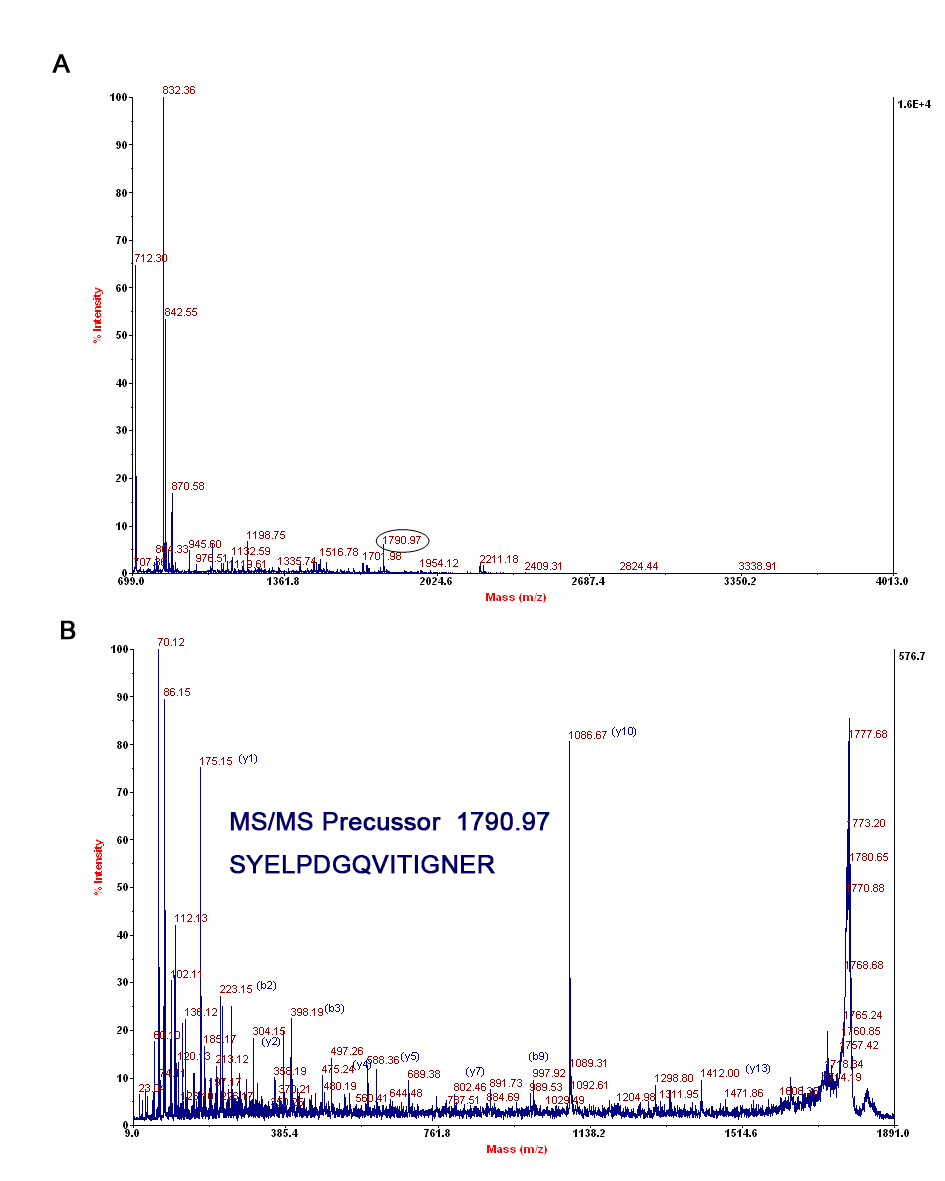

Supplement: Figure S3 — The MS and MS/MS spectra of β-actin. The figure displays the MS spectrum (A) and MS/MS spectrum marked with b ions and y ions (B) for β-actin identification. The sequence of precursor at m/z1790.97 was analyzed by MS/MS to be SYELPDGQVITIGNER. This protein was identified to be β-actin after database searching. (TIF) [file pone.0018834.s003.tif]

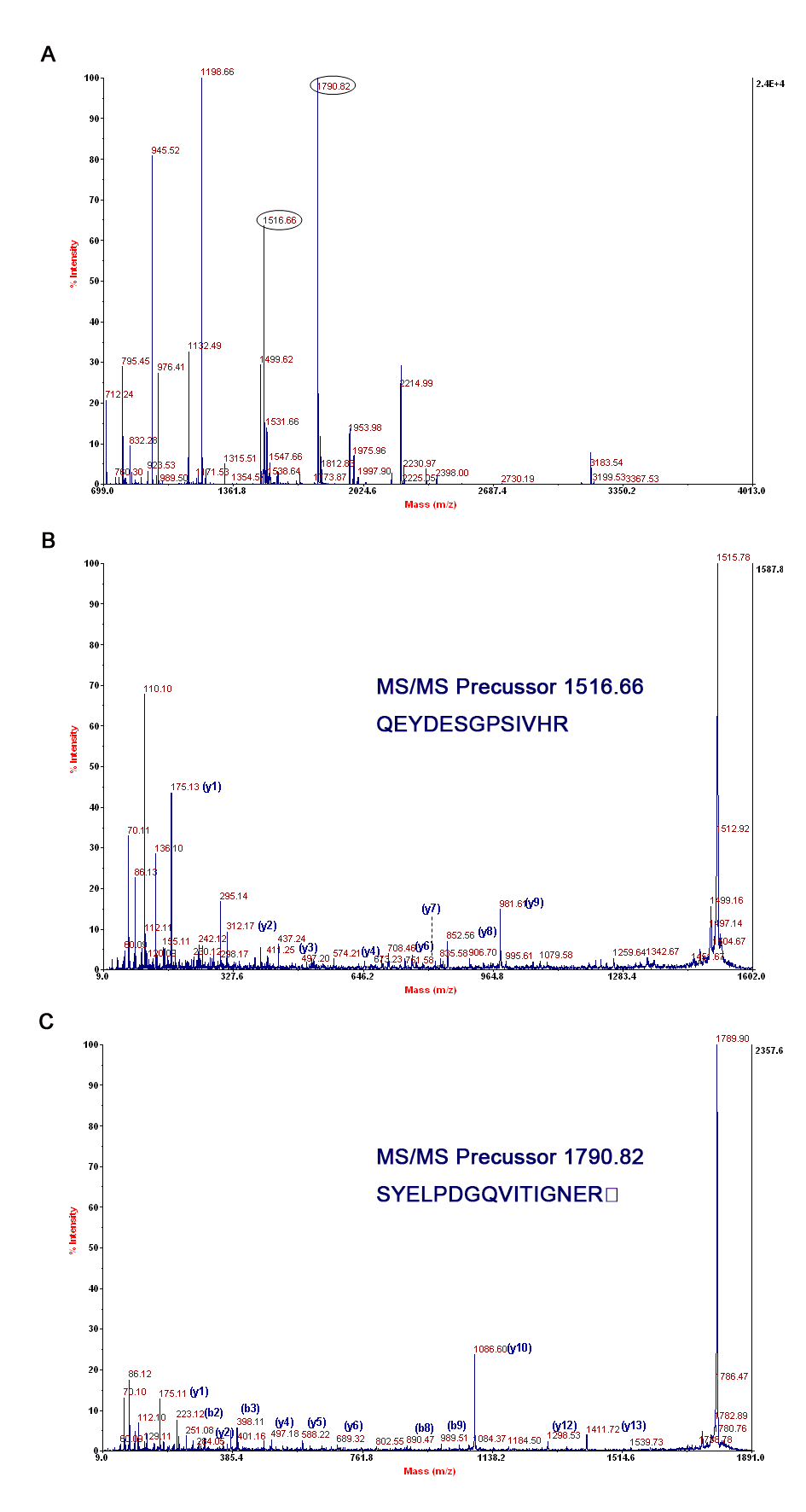

Supplement: Figure S4 — The MS and MS/MS spectra of γ-actin. The figure displays the MS spectrum (A) and two MS/MS spectra marked with b ions and y ions (B, C) for γ-actin identification. The sequences of precursor at m/z1516.66 and m/z1790.82 were analyzed by MS/MS to be QEYDESGPSIVHR and SYELPDGQVITIGNER, respectively. This protein was identified to be γ-actin after database searching. (TIF) [file pone.0018834.s004.tif]

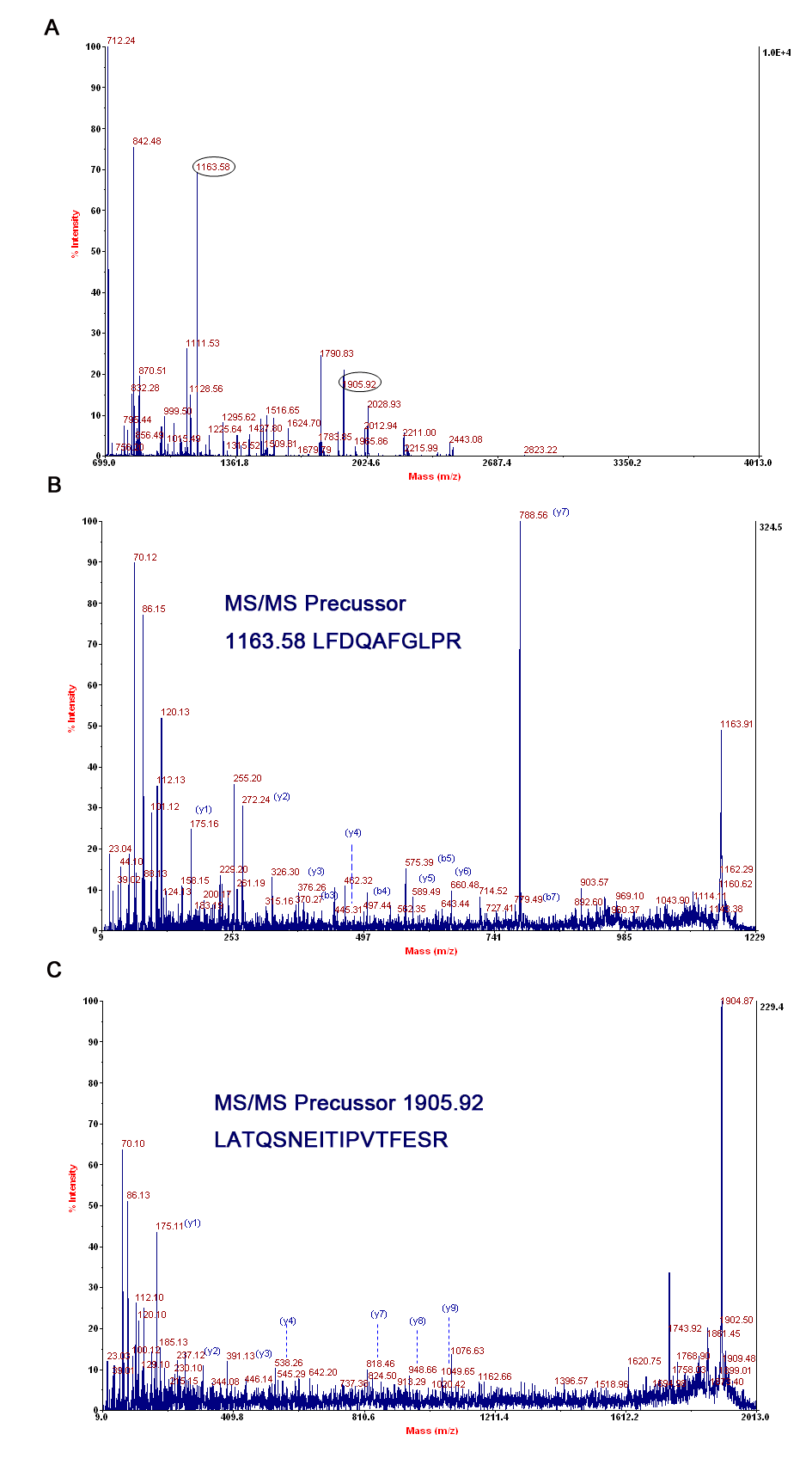

Supplement: Figure S5 — The MS and MS/MS spectra of HSP27. The figure displays the MS spectrum (A) and two MS/MS spectra marked with b ions and y ions (B, C) for HSP27 identification. The sequences of precursor at m/z1163.58 and m/z1905.92 were analyzed by MS/MS to be LFDQAFGLPR and LATQSNEITIPVTFESR, respectively. This protein was identified to be HSP27 after database searching. (TIF) [file pone.0018834.s005.tif]
